# Enhancing the Usability of Patient Monitoring Devices in Intensive Care Units: Usability Engineering Processes for Early Warning System (EWS) Evaluation and Design

**DOI:** 10.3390/jcm14093218

**Published:** 2025-05-06

**Authors:** Hyeonkyeong Choi, Yourim Kim, Wonseuk Jang

**Affiliations:** 1 Department of Medical Device Engineering and Management, Yonsei University College of Medicine, Seoul 06229, Republic of Korea; hyeonkyeong97@daum.net (H.C.); yrkim0224@yuhs.ac (Y.K.); 2 Medical Device Usability Research Center, Gangnam Severance Hospital, Yonsei University College of Medicine, Seoul 06230, Republic of Korea

**Keywords:** patient monitoring system, usability test, early warning score, human factor engineering

## Abstract

**Background/Objectives**: This study aimed to enhance the usability of patient monitoring systems by integrating the Early Warning Score (EWS) function and improving user interface elements. The EWS function is expected to enable the early detection of acute deterioration and prompt medical intervention, while the optimized design supports rapid decision-making by nursing staff. **Methods**: Two formative usability evaluations were conducted to identify user requirements and improve the device design. A simulated usability test involved five ICU medical staff members, followed by a user preference survey with 72 ICU staff members in a real clinical setting. After incorporating feedback, a summative usability test with 23 ICU nurses was performed to evaluate the revised device. **Results**: Issues related to unfamiliar parameter terminology and alarm message positioning were identified, and the need for the EWS function was emphasized. The summative evaluation showed an increase in task success rate from 86% to 90% and a significant improvement in user satisfaction from 74.85 (SD: 0.88) to 89.55 (SD: 0.75) (*p* < 0.05). **Conclusions**: The integration of the EWS function and interface improvements significantly enhanced the usability of patient monitoring system. These advancements are expected to enable rapid detection of patient deterioration and support timely clinical decision-making by ICU staff.

## 1. Introduction

A patient monitoring system is a medical device used in hospitals to continuously monitor vital signs in adult, pediatric, and neonatal patients [1]. These devices measure various physiological parameters, including electrocardiography (ECG), diagnostic electrocardiography (12 Lead ECG), heart rate/pulse rate (HR/PR), non-invasive blood pressure (NIBP), oxygen saturation (SpO_2_), invasive blood pressure (IBP), respiration, partial pressure of carbon dioxide (EtCO_2_, InCO_2_), temperature, and multi-gas monitoring [1]. They are utilized to monitor patients’ vital signs in diverse medical environments. Despite advancements in medical technology, the user interface of patient monitoring systems has remained largely unchanged for over 20 years, with layout and structural elements displaying minimal evolution [1]. Typically, patient monitoring systems are organized into waveform areas, numeric data displays, and a lower menu configuration bar. It is important to note that not all parameters are displayed as both waveforms and numerical values. Parameters such as ECG, SpO_2_, IBP, RESP, and EtCO_2_ are presented as both waveforms and numeric data simultaneously, whereas parameters like NIBP and temperature (TEMP) are displayed only as numeric values.

Patient monitoring systems are deployed in various clinical settings, including general wards, intensive care units (ICUs), and operating rooms [2]. In ICUs, patients are often connected to a range of medical equipment, including patient monitoring systems and ventilators, requiring continuous observation. Continuous patient monitoring plays a crucial role in critical care, serving as an essential device for promptly detecting changes in a patient’s condition [3]. Medical staff, primarily physicians and nurses working in ICUs, are the primary users of these devices. Due to the critical nature of patients requiring continuous monitoring, it is essential to regularly assess the measured parameters to promptly detect and respond to severe adverse events and potential risks [2].

The Early Warning Score (EWS) is a standardized assessment tool designed to facilitate the early detection of acute deterioration in hospitalized patients, enabling prompt medical intervention [4,5,6]. Introduced in 1997, EWS was developed to address the problem of delayed recognition of vital sign deterioration among ward patients [7]. It is effective in identifying ward patients at risk of cardiac arrest, death, or transfer to ICU [7]. The EWS system encompasses various scoring tools tailored to different patient populations, including the National Early Warning Score (NEWS), which is the standardized system adopted by the British National Health System (NHS), the Pediatric Early Warning Score (PEWS) designed for pediatric patients, and the Modified Early Warning Score (MEWS), which incorporates clinical parameters such as urine output, as shown in Table 1 [8,9,10].

The NEWS, developed in 2012, differs from conventional EWS scoring systems by including an oxygen saturation parameter, allowing for a more precise reflection of a patient’s respiratory status [9,11]. Prior to the implementation of NEWS, studies revealed that approximately 20% of hospitalized patients in general wards exhibited abnormal vital signs, with a threefold increase in the 30-day mortality rate compared to patients with normal vital signs [12,13]. The primary purpose of NEWS is to standardize clinical monitoring within wards and to support clinical judgment and decision-making by nurses [12]. Numerous studies have demonstrated that the use of EWS in ICUs is associated with reduced mortality rates, and monitoring with EWS has been shown to significantly decrease the incidence of adverse events post-discharge from the ICU [5,14,15]. The PEWS, specifically developed for pediatric patients, not only evaluates cardiovascular and respiratory status but also considers behavioral factors such as playability, sleep state, lethargy, and excretory functions, including urine and feces output [10]. This comprehensive approach aids in the early detection of clinical deterioration in pediatric patients [8].

By enabling nurses to quantitatively assess the clinical status of patients, EWS facilitates early warning and rapid response [12]. Therefore, to maximize the clinical effectiveness of EWS, it is essential to ensure the intuitiveness and usability of the user interface, as well as to enhance the clinical evaluation competence and system utilization capabilities of healthcare providers.

The user interface of medical devices must ensure intuitiveness and usability to support nurses in making rapid and clinically sound decisions [16,17]. To achieve this, a systematic approach that incorporates usability engineering is essential. Usability engineering is a process carried out throughout the entire lifecycle of medical device development, reflecting user needs from the design phase and continuously improving through iterative evaluations [17,18].

In both the Republic of Korea and Europe, usability engineering processes are conducted based on IEC 62366-1:2020, while in the United States, they follow guidelines established by the Food and Drug Administration (FDA), including the 2016 publications “Applying Human Factors and Usability Engineering to Medical Devices” and “Content of Human Factors Information in Medical Device Marketing Submissions” [19,20]. Usability engineering activities involve performing usability evaluations during the verification and validation stages, which consist of formative and summative evaluations.

Usability evaluation is defined as the process of assessing the usability and effectiveness of medical devices by intended users in the intended use environment [16,20,21,22,23]. Formative evaluation is primarily conducted in the early stages of medical device development, focusing on iterative assessments to incorporate user requirements into design improvements [20,21].

These evaluations are performed at various stages, from initial prototypes to final prototypes, and are conducted with a minimum of five users as specified by IEC TR 62366-2:2016 [20,21]. IEC TR 62366-2:2016 provides detailed guidance for formative evaluations, emphasizing that even small sample sizes can effectively uncover a significant proportion of usability issues [21]. According to the standard, if the probability of a user encountering a specific usability problem is 0.25, conducting a usability test with six participants results in an approximately 82% likelihood of detecting at least one occurrence of the issue [21]. This statistical principle supports the recommendation that involving five to eight users in formative evaluations can efficiently identify critical usability problems without requiring large sample sizes [21]. Consequently, formative evaluations are designed to be pragmatic and iterative, enabling timely design modifications based on feedback from a small number of early users.

Summative evaluation, on the other hand, is conducted at the final prototype stage of medical devices with at least 15 intended users to evaluate usability and ensure that user requirements identified during formative evaluations have been adequately addressed [20,21]. This evaluation verifies the usability of the final product and confirms whether the device meets the intended usability criteria.

The primary objective of this study is to enhance the usability of patient monitoring systems utilized in ICUs by conducting usability evaluations based on usability engineering activities. In particular, the study focuses on verifying the ease of use and efficiency of the integrated EWS function within patient monitoring systems. Through this approach, the study aims to propose a medical device design strategy that reduces the workload of medical staff in real-world settings while supporting prompt clinical decision-making.

## 2. Materials and Methods

### 2.1. Study Design

In this study, three usability evaluations were conducted, consisting of two formative evaluations and one summative evaluation. Table 2 presents the number of participants and evaluation metrics for each formative and summative evaluation conducted in this study.

The first formative evaluation involved actual use testing in a real environment, targeting ICU nurses who utilized the target device (M50, Mediana Co, Gangwon-do, Republic of Korea) alongside a control device (MP70/MX700, Philips Medical Systems, Andocer, MA, USA). A survey was conducted to gather feedback on usability. For each scenario, participants evaluated the system based on three key dimensions: Usefulness, Ease of Use, and Satisfaction [24]. In this study, the USE Questionnaire (Usefulness, Satisfaction, and Ease of Use), a representative instrument developed by Arnie Lund to assess the usability of products and systems, was employed [24]. This questionnaire is particularly suitable for evaluating software and digital health systems and is frequently used to compare usability before and after user interface (UI) improvements [25]. The evaluation items were organized according to the characteristics of each scenario and categorized into the three dimensions accordingly [24,25]. The detailed questionnaire items are provided in Supplementary Table S1. The second formative evaluation was carried out in a simulated environment with ICU medical staff, using a dummy patient to perform a usability test.

The summative evaluation was conducted with ICU nurses from the cardiology intensive care unit, also in a simulated environment, using the evaluation device. The usability test required the presence of a test administrator, data analyst, and simulator operator. The test administrator guided the evaluation process, while the data analyst recorded and documented the evaluation procedures from an observation room. Surveys were administered based on the test administrator’s instructions, and participants completed the survey items accordingly. In the summative evaluation, the survey was structured based on the same three dimensions applied in the formative evaluation: Usefulness, Satisfaction, and Ease of Use. It was administered in alignment with the specific characteristics of each scenario. The detailed survey items are provided in Supplementary Table S2.

Following the two formative evaluations, user requirements from the usability perspective were reflected in the evaluated device. In particular, adjustments were made, such as modifying the terminology of parameters within the patient monitoring systems and adding functions, including the EWS feature. Subsequently, the usability of the improved medical device was evaluated, with a particular focus on assessing the necessity and usability of the newly added EWS function in the actual ICU setting.

### 2.2. Participants

The participants of this study were recruited from among medical staff, who are the primary intended users of patient monitoring systems. During the formative evaluation, the usability test conducted in a simulated environment was performed with five medical staff members from Severance Hospital, following the participant recruitment criteria outlined in IEC TR 62366-2:2016 [21]. For the actual use evaluation conducted in a clinical environment, a survey was administered to 72 nurses from Severance Hospital.

In accordance with the international standard IEC TR 62366-2:2016 if the probability of a user encountering a usability issue for a specific task is assumed to be 0.25, the likelihood of detecting at least one occurrence of the issue is approximately 76% with five participants and increases to 99% with fifteen participants [21]. In formative evaluations, repeated participation by the same users enables the effective identification of usability problems, even with a small sample size [21]. Furthermore, in accordance with the recommendation of IEC TR 62366-2:2016 that summative usability evaluations include at least 15 participants, this study conducted the summative evaluation with 23 nurses from Severance Hospital [20,21]. All participants were nurses with more than one year of experience in using patient monitoring systems.

This study was approved by the Institutional Review Board (IRB) of Severance Hospital, Yonsei University Health System, and was conducted in compliance with all relevant guidelines and regulations related to research methodology. Prior to participation, informed consent was obtained from all participants, ensuring ethical conduct throughout the usability evaluation process. (Usability test: No. 3-2022-0270, User preference survey: No. 1-2021-0030, No. 9-2021-0080).

### 2.3. Study Procedures

#### 2.3.1. Formative Evaluation

- Usability Test

The formative evaluation was conducted individually with five ICU medical staff members, each participating in the evaluation environment independently. To facilitate the assessment, task-based scenarios were developed to reflect the typical workflow of patient monitoring systems. Each participant performed the evaluation under the guidance of the test administrator. To ensure consistency, the test administrator provided standardized instructions solely to clarify the scenario objectives and did not intervene in participants’ task execution or decision-making process. This approach was adopted to minimize potential methodological bias related to facilitator influence. During the evaluation, the simulator operator manipulated the patient’s vital sign values according to pre-defined variation patterns within the simulated scenario, thereby creating a situation closely resembling actual clinical settings.

The data analyst observed and recorded the evaluation process remotely from the observation room using Blackmagic Media Express software (version 1.0), ensuring comprehensive data collection and analysis.

**Figure 1:**
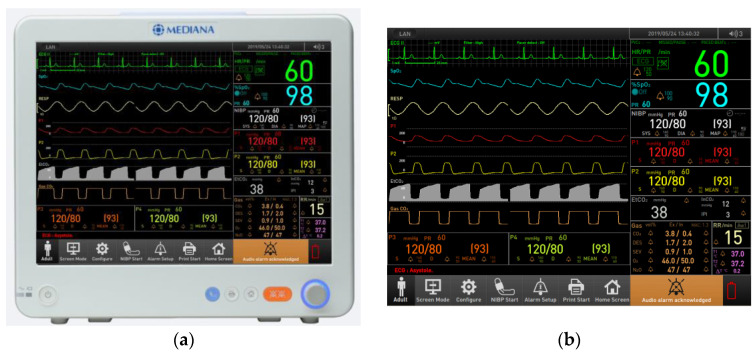
Mediana patient monitoring system (M50) during formative evaluation: (**a**) system; (**b**) main screen.

Prior to the usability evaluation, participants were provided with pre-training on the purpose of the evaluation and the correct usage of the device. The target device for evaluation was the Mediana patient monitoring system (M50), as shown in Figure 1. The main screen of the device is divided into four primary areas: the waveform area displaying seven waveforms, the parameter area presenting 11 numeric data points, the menu button section, and the alarm message area located at the bottom of the main screen.

To prevent any direct influence from prior training on the evaluation process, a 15 min interval was maintained between the training session and the evaluation. During the evaluation, participants performed 6 scenarios, encompassing the entire workflow of using the patient monitoring system from patient admission to discharge, with a total of 40 tasks. A detailed description of these scenarios and tasks is summarized in Table 3.

The evaluation environment was carefully designed to replicate the actual usage environment of the patient monitoring system, specifically the ICU. The setup included a hospital bed, a patient dummy, and other necessary medical equipment to create a realistic clinical setting. Additionally, to closely mimic the real ICU environment, the evaluation room temperature was maintained between 21 °C and 24 °C, while the humidity was controlled within the range of 30% to 60% prior to the commencement of the evaluation.

- User preference survey

The usability evaluation was conducted in an actual clinical setting, specifically in ICU of Severance Hospital. During the evaluation, participants utilized both the target device (M50, Mediana Co, Gangwon-Do, Republic of Korea) and the control device (MP70/MX700, Philips Medical Systems, Andover, MA, USA) to monitor patients’ vital signs. Following the monitoring process, a preference survey was administered to identify potential design improvements for the target device.

The survey was structured to include alarm items associated with high-risk factors, additional functions deemed necessary, and elements requiring usability enhancement. Through this approach, the study aimed to analyze the user experience of the target device and derive practical improvement strategies applicable to the clinical environment.

#### 2.3.2. Summative Evaluation

The summative evaluation was conducted to assess the usability of the EWS function, which was newly incorporated during the design stage following the formative evaluation. To accommodate the inclusion of the EWS function, additional scenarios were developed based on the existing formative evaluation tasks. The evaluation method employed was consistent with that of the formative evaluation, utilizing a usability testing approach. A total of 23 participants took part in the assessment, and specific EWS-related tasks are presented in Table 4.

To comprehensively evaluate the usability of the EWS function, diverse scenarios were constructed, including modifying EWS protocols, calculating EWS scores, verifying EWS messages, and reviewing EWS trend data. Like the formative evaluation, each participant individually performed the evaluation under the guidance of the test administrator. During the evaluation, the simulator operator adjusted the vital sign parameters of simulated patients to create various clinical scenarios. For the physiological parameters related to the EWS assessment, values were provided via the simulator. In contrast, the level of consciousness was designed to be manually entered by the user, depending on the task scenario. The level of consciousness was assessed using the AVPU scale (Alert, Verbal, Pain, Unresponsive), which allows for a rapid and straightforward evaluation, and was implemented in the system through selectable input options. Additionally, the data analyst monitored the entire evaluation process from the observation room through a one-way mirror, meticulously recording all interactions made by the participants.

### 2.4. Analysis

In this study, the usability tests conducted during both formative and summative evaluations were analyzed by calculating the success rate of each task, categorized as completed, completed with issues, or non-completed. During the formative evaluation, a satisfaction assessment was performed using a 7-point Likert scale, and the mean and standard deviation for each scenario were calculated. In the summative evaluation, satisfaction was assessed using a 5-point Likert scale, and the mean and standard deviation were calculated in the same manner as the formative evaluation. In survey-based research, 5-point and 7-point Likert scales are among the most frequently utilized formats [26]. The 7-point Likert scale provides enhanced response granularity, thereby enabling the capture of more nuanced perceptions and subtle distinctions in user feedback [26,27]. Accordingly, the formative evaluation in this study employed a 7-point scale to facilitate the collection of detailed usability insights. In contrast, the summative evaluation adopted a 5-point Likert scale to reduce cognitive burden on respondents and to ensure a more streamlined and cognitively efficient response process. For the preference survey, items with yes-or-no responses were calculated as the proportion of each response. Since the satisfaction assessments used different Likert scales between the formative and summative evaluations, scores were converted to a 100-point scale to enable comparison of satisfaction levels. The analysis of satisfaction scores, preference evaluations, task success rates from formative evaluation, and task success rates from summative evaluation was performed using SPSS version 27 (IBM Corp., Armonk, NY, USA). To compare satisfaction scores between formative and summative evaluations, the Mann–Whitney U test was employed. As the participants in the formative and summative evaluations constituted two mutually exclusive groups of nurses, the assumption of independence between the two samples was satisfied. Given that the sample size was less than 30, a test for normality was performed. The results indicated a violation of the normality assumption (*p* < 0.05). Accordingly non-parametric statistical analyses were applied. Descriptive statistics were presented as mean and standard deviation, and statistical significance was set at *p* < 0.05. User preference results were expressed as percentages for each item.

## 3. Results

### 3.1. Execution of the Formative Evaluation

#### 3.1.1. User Statistics of the Formative Evaluation

The formative evaluation was conducted in two phases: a usability test performed in a simulated environment and a survey conducted in a real clinical environment. Demographic information of the participants is presented in Table 5. The evaluation participants consisted of medical staff working in the ICU at Severance Hospital.

For the usability test conducted during the formative evaluation, the average age of participants was approximately 39.6 years, and the average clinical experience was 15.4 years. In contrast, according to the survey conducted in the clinical environment, the average age of participants was approximately 30.8 years, with an average clinical experience of 7.6 years.

#### 3.1.2. Task Completion of the Formative Evaluation

In this study, a total of 40 detailed tasks were performed across six usability scenarios, as shown in Table 3. The success rates for each scenario are presented in Table 6, indicating that participants achieved an average success rate of 86%. The scenario with the highest success rate was related to patient discharge, while the scenario with the lowest success rate was associated with general settings, which recorded a success rate of 73%.

Within each scenario, detailed tasks were classified into critical tasks and non-critical tasks based on their severity. Notably, critical tasks included settings related to waveforms and parameters (Task 4) and alarm settings (Tasks 13–32). Although all scenarios demonstrated a success rate of 70% or higher, there were notable differences in success rates between scenarios (Table 6).

Among the critical tasks in which failures were observed, Task 4 (“Change the label from PAP to P2”) within the waveform/parameter setting scenario presented notable challenges (see Table 6). This difficulty is attributed to the fact that the terms “P1” and “P2” are not commonly used in clinical practice, which likely caused confusion among users and adversely affected task performance. Even participants who successfully completed the task reported unfamiliarity with the terminology. Similarly, in the general alarm setting scenario, Task 18 involved changing the upper systolic alarm limit for P1, but participants faced similar difficulties in locating the parameter due to unfamiliarity with the terminology. Consequently, the task of adjusting the upper limit could not be completed. These findings indicate that unfamiliar terminology within critical tasks can significantly hinder usability, even among experienced medical staff.

A satisfaction survey was conducted to assess the ease of use for each scenario, and Table 7 presents the mean satisfaction scores along with the standard deviations. The overall mean satisfaction score across all scenarios was 5.49 (standard deviation: 0.88). The scenario with the highest satisfaction score was related to patient discharge, with a score of 7.00.

An analysis of satisfaction scores for tasks with low success rates due to unfamiliar terminology revealed that the item related to parameter terminology received a score of 5.73 ± 0.88. Although the task of changing parameter labels itself was relatively easy, the unfamiliarity with abbreviations and terminology negatively impacted both the task success rate and satisfaction score.

The formative evaluation aimed to identify areas for improvement by analyzing task success rates, failure rates, and observed usability errors. Additionally, feedback from satisfaction surveys was incorporated to propose enhancements to the evaluated equipment.

#### 3.1.3. User Preference Survey

**Figure 2:**
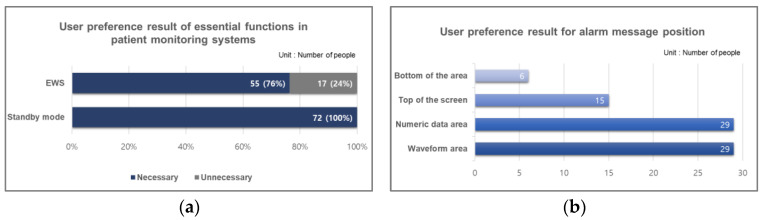
User preference result: (**a**) necessity assessment of EWS and standby mode functions; (**b**) user preference analysis on alarm message position.

This study conducted a preference survey involving 72 participants who utilized both the Mediana device and the Philips device to assess preferences regarding patient monitoring systems. The results are presented in Figure 2.

Figure 2a illustrates the results of the preference survey regarding additional functionalities required for the patient monitoring system, with the aim of identifying user requirements for the evaluated device. Approximately 76% (55 participants) responded that EWS function is necessary, as it provides critical signals indicating the deterioration of a patient’s condition. Additionally, the preference survey on the necessity of the standby mode function revealed that 100% (72 participants) deemed it essential. Participants who had experience using both devices indicated that the Philips device already includes the standby mode function, which is frequently utilized during patient transport, emphasizing the need for this functionality in the Mediana patient monitoring system (M50) as well.

Figure 2b shows the results of the preference survey related to alarm settings on patient monitoring systems. The preference for the alarm message location allowed for multiple selections. Participants predominantly preferred the alarm message to be displayed within the parameter area and the parameter value area on the screen. Additionally, there was a notable preference for displaying alarm messages in the upper area of the screen. These findings indicate that the EWS function and standby mode are perceived as critical features by users, and improvements in the visual placement of alarm messages can enhance usability and user satisfaction.

### 3.2. Design Implementation

Through two rounds of formative evaluations, the user interface (UI) of the patient monitoring system was improved by incorporating user requirements. As shown in Figure 2a, 76% of ICU nurses indicated the need for EWS function, leading to its incorporation into the UI.

**Figure 3:**
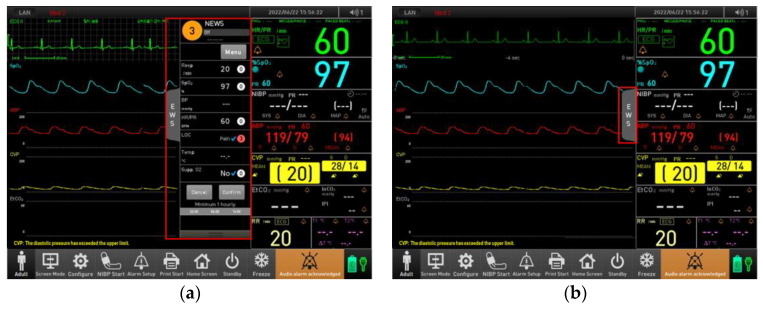
Main screen with EWS (Early Warning Score) functionality: (**a**) activated; (**b**) deactivated.

**Figure 4:**
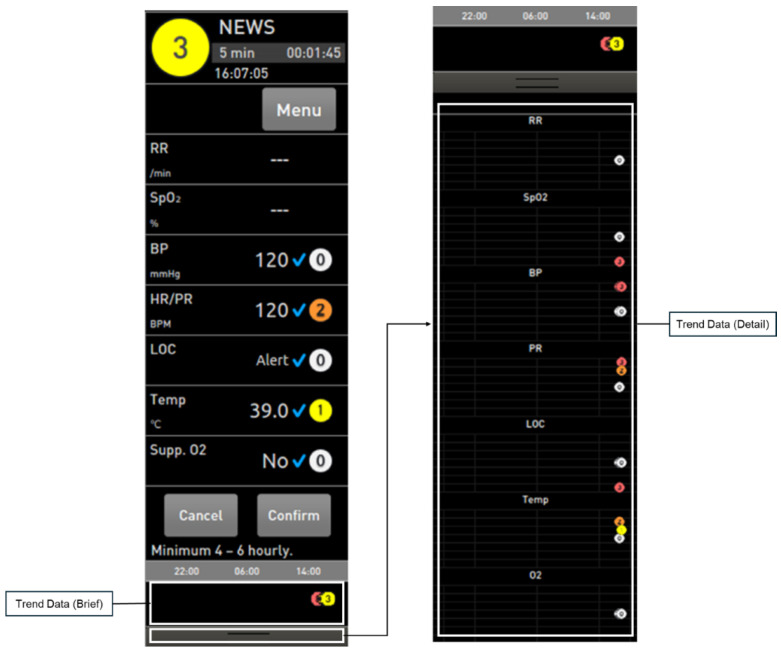
Detailed view and data trends of EWS (Early Warning Score).

As shown in Figure 3 and Figure 4, when the user presses the “Calculate” button within the EWS area, the resulting score is displayed at the top of the screen. Additionally, detailed information on the EWS trend can be accessed via the black bar at the bottom of the screen. Once the EWS score is saved, users can view detailed scores for each parameter. The score is categorized into four levels based on risk severity: Standard (White), Low (Yellow), Intermediate (Orange), and High (Red). When applying the National Early Warning Score (NEWS) protocol, the total score is calculated based on seven physiological parameters: respiratory rate, oxygen saturation, systolic blood pressure, heart rate/pulse, level of consciousness, body temperature, and use of supplemental oxygen. The level of consciousness is evaluated using the AVPU scale, which allows for the selection of one of four categories: Alert, responsive to Verbal stimuli, responsive to Pain, or Unresponsive. The EWS feature can be activated as shown in Figure 3a, allowing it to be displayed on the main screen when in use. When the EWS feature is not in use, it can be configured to appear as shown in Figure 3b within the main screen. By selecting the EWS option in Figure 3b, the display expands to resemble the layout shown in Figure 3a. Additionally, within the parameter settings, it is possible to configure the system to completely hide the EWS feature, thereby optimizing screen space and user interface according to clinical preferences.

To enhance user convenience, the system was designed to automatically calculate scores based on the parameter value ranges, thereby minimizing manual input and reducing the cognitive burden on users.

**Figure 5:**
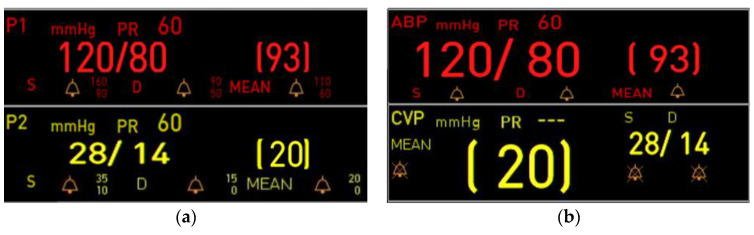
Parameter terminology modification screen: (**a**) user interface displaying parameter terminology (P1, P2) during the formative evaluation; (**b**) enhanced user interface displaying parameter terminology (ABP, CVP) during the summative evaluation, reflecting improvements in terminology clarity post-formative evaluation.

To improve the high failure rate observed in critical tasks, the terminology used for parameters was revised. As shown in Figure 5a, the original parameter terms (P1, P2) were removed and replaced with clinically familiar terms such as ABP (Arterial Blood Pressure), CVP (Central Venous Pressure) in Figure 5b. This modification was implemented to enhance intuitiveness by adapting the interface with terminology commonly used by medical staff.

**Figure 6:**
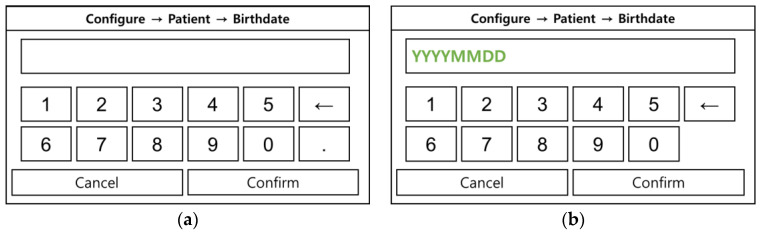
Patient registration screen: (**a**) user interface for date of birth entry in patient registration during the formative evaluation; (**b**) improved user interface for date of birth entry in patient registration during the summative evaluation, reflecting UI enhancements post-formative evaluation.

Although patient registration is not considered a highly critical task, usability improvements were implemented to reduce confusion. In the previous date of birth entry format shown in Figure 6a, the “.” separators were removed. Additionally, as shown in Figure 6b, a prompt indicating the input format as “YYYYMMDD” was added to the entry field. This modification was designed to minimize input errors and enhance user convenience by providing clear guidance during data entry.

### 3.3. Execution of the Summative Evaluation

#### 3.3.1. User Statistics of the Summative Evaluation

A total of 23 participants took part in the summative evaluation, consisting of nurses working in the cardiac intensive care unit (CCU) at Severance Hospital. The usability test was conducted to evaluate the suitability of the device in a clinical setting. Table 8 presents the demographic statistics of the participants involved in the summative evaluation.

#### 3.3.2. Task Completion of the Summative Evaluation

The summative evaluation was conducted using 9 scenarios comprising a total of 58 detailed tasks to assess usability. The scenarios used in the summative evaluation are presented in Table 9. They were developed by extending the original formative evaluation scenarios with the addition of new scenarios focusing on the EWS function, wave freeze, and standby mode. As shown in Table 9, Participants achieved an average success rate of 90%. Notably, EWS function, which was newly introduced after the formative evaluation, demonstrated a scenario success rate of 82%.

The task with the lowest success rate within the EWS scenarios involved verifying the total EWS score and message. In this task, most participants successfully identified the total score displayed at the top of the EWS screen on the main interface but failed to locate the message displayed at the bottom of the EWS menu. Participants reported that the text size of the message was too small and lacked sufficient visibility, making it difficult to notice. The second lowest success rate was observed in the task of verifying trend data, including the total EWS score. Specifically, four participants were unable to locate the EWS trend data, while two participants attempted to check the data within the event review outside of the EWS screen. Additionally, two participants managed to identify the trend data location but failed to recognize the overall trend of the total score displayed through the black bar at the bottom of the trend screen. This issue appears to stem from participants’ unfamiliarity with the method of accessing the total trend data through the black bar, as it was a function they had not previously used. This lack of prior experience with the feature likely contributed to difficulties in completing the task successfully.

### 3.4. Comparative Usability Analysis Between Formative and Summative Evaluations

**Figure 7:**
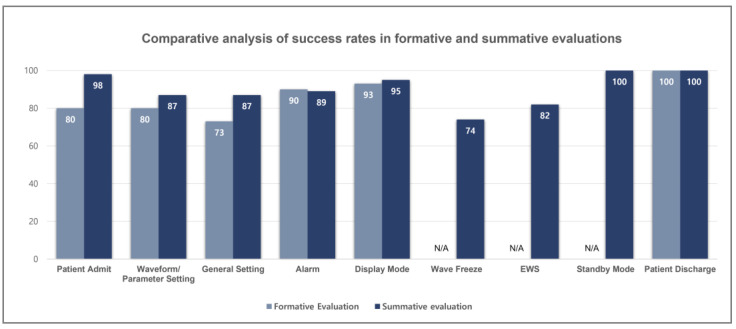
Comparative analysis of success rates in formative and summative evaluations across each scenario.

In this study, statistical analyses were conducted on task success rates and satisfaction survey data obtained through usability evaluations to assess the impact of design modifications implemented after the formative evaluation on subsequent summative evaluation outcomes. Scenario-specific task performance was compared between the two evaluation phases, as illustrated in Figure 7. The analysis demonstrated that task success rates improved across most scenarios in the summative evaluation relative to the formative evaluation.

The formative evaluation was conducted with five ICU medical staff, whereas the summative evaluation involved a separate group of 23 medical staff from the same institution. Normality of the task success rate and satisfaction data was assessed using the Shapiro–Wilk test, which revealed a violation of the normality assumption (*p* < 0.05). Accordingly, nonparametric Mann–Whitney U tests were employed for subsequent statistical comparisons. The results are presented in Table 10.

Following the formative evaluation, device modifications were implemented with a focus on addressing usability issues identified in low-performing and high-priority tasks where participants had expressed significant concerns. Differences in task success rates across all scenarios were not statistically significant (*p* > 0.05). In the “Waveform/Parameter Setting” scenario, where terminology revisions were applied, the success rate increased from 80% in the formative evaluation to 87% in the summative evaluation. The Mann–Whitney U test yielded a *p*-value of 0.071, which did not reach the conventional threshold for statistical significance (*p* < 0.05); therefore, no statistically significant difference was identified.

**Figure 8:**
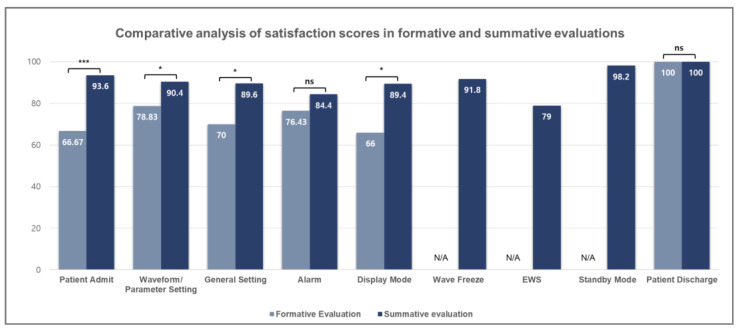
Comparative analysis of satisfaction scores in formative and summative evaluations across each scenario. Significance levels are indicated as follows: * *p* < 0.05, *** *p* < 0.001, ns = not significant, based on the *p*-values in Table 11.

To compare satisfaction levels, evaluation scores were converted to a 100-point scale and analyzed across all scenarios. The results, illustrated in Figure 8, revealed consistent increases in satisfaction scores following design improvements. To statistically assess these changes, Mann–Whitney U tests were performed using SPSS, with detailed results presented in Table 11.

One of the key improvements involved changing parameter terminology, specifically within the waveform and parameter setting scenario. Analyzing the satisfaction scores before and after the improvement revealed a *p*-value of 0.027, which is less than 0.05, indicating a statistically significant increase in satisfaction from the formative evaluation (mean score: 78.83) to the summative evaluation (mean score: 90.4).

Additionally, device improvements were made to address the low task success rate and satisfaction during patient registration, particularly regarding the data entry method. A comparative analysis of satisfaction scores before and after the improvement yielded a *p*-value of 0.001, which is also less than 0.05, indicating a significant improvement in satisfaction from the formative evaluation (mean score: 66.67) to the summative evaluation (mean score: 93.6).

## 4. Discussion

The summative evaluation was conducted to assess the improved patient monitoring systems following the user interface enhancements made after the formative evaluation. The comparison of task success rates between the formative evaluation and summative evaluation is presented in Figure 7. The task success rate increased from 86% in the formative evaluation (usability test) to 90% in the summative evaluation (usability test). An improvement in success rates was observed across most scenarios, particularly for the newly added functions, such as EWS, wave freeze, and standby mode.

In the context of the domestic clinical environment, the EWS function is not widely utilized, resulting in limited direct experience among nurses. Although the summative evaluation exhibited a relatively lower success rate compared to other scenarios, this outcome was attributed to the lack of prior user experience with the function. Interviews conducted with participants who experienced difficulties using the EWS function revealed that they believed the system could be used smoothly with adequate training and accumulated experience.

Considering the user requirements derived from the formative evaluation, it is evident that the EWS function holds significant potential in supporting proactive responses by enabling nurses to preemptively check patient information within the complex ICU environment. Furthermore, the EWS function is particularly beneficial in general wards, where the necessity of monitoring patients’ conditions in advance is crucial for early detection of acute deterioration. In general wards, where monitoring frequency is lower and direct observation medical staff is limited, the EWS function can serve as a critical tool for identifying potential risks at an early stage.

In the case of patient management, the scenario success rate increased from 80% to 98%, while the satisfaction score improved from 66.67 to 93.6. Although the change in task success rate was not statistically significant (*p* = 0.239), the improvement in satisfaction was highly significant (*p* = 0.001). The observed difference in success rates appears to be due to the differing number of participants in the formative and summative evaluations, despite the same number of failures in both groups. These results indicate that even with high success rates, user satisfaction regarding ease of use was further enhanced through device design improvements, highlighting the impact of usability-focused refinements.

In the waveform and parameter setting scenario, the success rate increased from 80% in the formative evaluation to 87% in the summative evaluation; however, this difference did not reach statistical significance (*p* = 0.071). In contrast, the satisfaction score significantly improved from 78.83 to 90.4, with a *p*-value of 0.027. Taken together, these results indicate that revising the interface using terminology familiar to clinical practice contributed to increased user satisfaction with waveform and parameter configuration. This finding suggests that ensuring consistency and intuitiveness in terminology can reduce cognitive load and enhance the overall user experience in medical device interfaces.

The success rate related to alarms decreased slightly from 90% in the formative evaluation to 89% in the summative evaluation. However, satisfaction scores improved significantly from 76.43 points in the formative evaluation to 84.40 points in the summative evaluation. Alarm functionality is crucial in ICU, as it enables the detection of potential risks based on patients’ vital signs. Similarly, EWS serves as a proactive measure to reduce alarm occurrences by identifying patient deterioration at an early stage.

Regarding patient discharge, both the success rate and satisfaction score remained consistently high, with 100% success rates and 100 points of satisfaction observed in both evaluations. This outcome indicates that, despite changes in participants and evaluation methods between the formative and summative evaluations, all users consistently found the user interface related to patient discharge to be intuitive and useful.

These results suggest that device improvements had a substantial and positive impact on user experience and satisfaction, reinforcing the effectiveness of addressing usability issues identified during the formative evaluation. The findings of this study suggest that device improvements made after the formative evaluation had a significant positive impact on user satisfaction. Furthermore, the process of deriving and applying user-centered improvement measures plays a pivotal role in enhancing the intended user experience. This underscores the importance of iterative usability evaluation in achieving high-quality, user-friendly medical devices.

## 5. Conclusions

This study aimed to enhance the usability of the patient monitoring system by integrating EWS function and improving the user interface. Through formative and summative evaluations, it was confirmed that iterative usability assessments significantly enhance device usability. The introduction of the EWS function is expected to facilitate early detection of acute patient deterioration, enabling prompt clinical intervention and supporting rapid decision-making by nurses.

However, this study has a few limitations. The usability evaluation was conducted exclusively with nurses from Severance Hospital, which limits the generalizability of the findings to various clinical settings and nurses. Future research should include a broader range of user groups and diverse clinical environments to ensure the universal applicability of the results.

## Figures and Tables

**Table 1:** Types of EWS (Early Warning Score).

	NEWS (National Early Warning Score)	MEWS (Modified Early Warning Score)	PEWS (Pediatric Early Warning Score)
Target patients	Adult patient	Adult patient	Pediatric patient
Purpose	Standardized early warning and tracking	Early detection of deterioration	Detect deterioration in pediatric patients
Standardization	Standardized in NHS, UK	Not standardized	Not standardized
Assessment Parameters	Respiration rate Oxygen saturation Systolic blood pressure Pulse rate Temperature Level of consciousness or new confusion (AVPU: Alert, Voice, Pain, Unresponsive)	Respiratory rate Blood pressure Heart rate Temperature Mental status Urine output	Respiratory rate Heart rate Blood pressure Oxygen saturation Temperature Level of consciousness Capillary refill time
Predictive Performance and Research Findings	Highly predictive in studies	Relatively low predictive power with variations across hospitals.	Useful for pediatric patients but lacks standardization.
Clinical Applications	Integration of emergency department, inpatient ward, and in-hospital patient monitoring with rapid response systems	Evaluation of critically ill patients within the hospital and criteria for ICU transfer.	Early detection of deterioration in pediatric emergency departments and pediatric wards

**Table 2:** Comparison of usability evaluation types: formative vs. summative evaluation.

	Formative Evaluation		Summative Evaluation
	Usability Test	Survey	Usability Test
Description	Scenario Execution: Participants perform assigned tasks in a simulated ICU environment, guided by the test administrator, and task completion is assessed. Satisfaction Survey: Evaluated using a 7-point Likert scale (1: Very Dissatisfied, 7: Very Satisfied) for each item.	Survey Execution: Participants complete a survey after using the target device (Mediana) and the comparator device (Philips) in a real ICU environment.	Scenario Execution: Participants perform assigned tasks in a simulated ICU environment, guided by the test administrator, and task completion is assessed. Satisfaction Survey: Evaluated using a 5-point Likert scale (1: Very Dissatisfied, 5: Very Satisfied) for each item.
Number of participants	5	72	23
Performance metric	Task completion assessment for 6 scenarios and 40 tasks categorized into three groups: completed (C), completed with issues (CI), not completed (NC) Ease of use, Usefulness, satisfaction: each scenario consists of 6 items	Preference Survey: three items with YES/NO responses and one item allowing multiple selections for preferred methods.	Task completion assessment for 9 scenarios and 58 tasks categorized into three groups: completed (C), completed with issues (CI), not completed (NC) Ease of use, Usefulness, satisfaction: each scenario consists of 9 items

**Table 3:** Use scenarios for formative evaluation of the patient monitoring system.

Use Scenario	Task No.	Task
Patient Admit	Task 1	Admit new patient.
	Task 2	Change the birthdate from 29 July 1970 to 20 July 1970.
Waveform/ Parameter Setting	Task 3	Change 4 channel waveform display in the standard mode.
	Task 4	Change the label from PAP to P2.
	Task 5	Change the waveform from Respiration to PAP.
	Task 6	Select ECG sweep speed to proper speed.
	Task 7	Select ECG waveform size menu to proper size.
	Task 8	Set NIBP interval to 1 h.
	Task 9	Change Respiration source randomly.
General Setting	Task 10	By default, the settings window closes after a certain amount of time. Change the settings so that the settings window does not close.
	Task 11	Set not to make a sound when the screen is touched.
	Task 12	Set the sound to be muted according to your heart rate.
Alarm	Task 13	Set the Alarm limit display ON to display established alarm value on the monitor. Check the alarm limit location on the screen and point it out.
	Task 14	Check all areas where the current visual alarm (SpO_2_) occurs.
	Task 15	Pause the alarm when the alarm occurs.
	Task 16	Adjust the lower threshold for the SpO_2_ alarm to 85%.
	Task 17	Check all areas where the current visual alarm (P1) occurs.
	Task 18	Change the upper limit of P1 SYS to 170 mmHg.
	Task 19	Check the alarm message in Message List and check the alarm message on the screen.
	Task 20	Set only the diastolic pressure and mean pressure auditory alarms of P1 to be disabled.
	Task 21	Set the arrhythmia alarm ON to display.
	Task 22	Change the alarm condition for ventricular tachycardia to 135 bpm.
	Task 23	Check the alarm of high-risk V-FIB and point to the area where the visual and alarm messages occur.
	Task 24	Pause the alarm when the alarm occurs.
	Task 25	Check the visual and audible alarm for a medium-risk Tachycardia alarm.
	Task 26	Check the visual and audible alarm for the low-risk Bigeminy alarms.
	Task 27	Check that the current ECG waveform is normal and click the message list to check the arrhythmia alarms that have occurred so far. (V-FIB alarm record does not remain)
	Task 28	Check a visual alarm to the current Pair PVCs alarm.
	Task 29	Check ECG waveform and PVCs alarm message list.
	Task 30	Check the visual alarm messages for new Run of PVCs alarms and compare them to ECG waveforms.
	Task 31	Check that the ECG waveform is Multiform PVCs and that the alarm message properly occurred. If the visual alarm message is not appropriate, wait for Multiform PVCs to appear.
	Task 32	The ECG waveform returned to normal. However, visual and audible alarms for Multiform PVCs are still occurring. Turn off the current alarm by pressing the button indicating that you have recognized Multiform PVCs.
	Task 33	Turn off all audible alarms.
Display Mode	Task 34	Display the Big Number mode.
	Task 35	Display the Tabular Trend mode.
	Task 36	Tabular trend list is ordered by descending now. Change the display order from ascending to descending.
	Task 37	Display the Graphical Trend mode.
	Task 38	Change the display interval randomly.
	Task 39	Display the Event review mode.
Patient Discharge	Task 40	Discharge the patient.

**Table 4:** Summative evaluation scenarios for the early warning score (EWS) function of the patient monitoring system.

Use Scenario	Task No.	Task
EWS (Early Warning Score)	Task 1	Configure the EWS Trend setting to NEWS2.
	Task 2	Set the AVPU scale in the EWS to PAIN.
	Task 3	Calculate the total EWS score.
	Task 4	Update the AVPU scale in the EWS to Alert
	Task 5	Recalculate the total EWS score.
	Task 6	Display the total EWS score along with the corresponding message
	Task 7	Review the trend data, including the total EWS score
	Task 8	Modify the system settings to hide the EWS pop-up bar, ensuring that EWS is not visible on the main screen.

**Table 5:** Sociodemographic characteristics and experience of the formative evaluation participants.

Variable		Usability Test	Survey
Age	20–29 years	0	30
	30–39 years	2	40
	40–49 years	3	2
Affiliated hospital	Severance Hospital	5	72
Work experience	Less than 3 years	0	1
	More than 3 years, less than 5 years	0	17
	More than 5 years, less than 10 years	2	38
	More than 10 years	3	16
Use experience with similar devices	Less than 3 years	0	1
	More than 3 years, less than 5 years	1	17
	More than 5 years, less than 10 years	1	38
	More than 10 years	3	16
Manufacturer name	Mediana	2 ^1^	72 ^1^
	Phillips	5 ^1^	72 ^1^

^1^ Repetition is acceptable.

**Table 6:** Success rate of each scenario in the formative evaluation (usability test).

Use Scenario	Success Rate	Comments
Patient Admit	80%	(Task 1,2) During the date of birth entry, a period “.” is automatically inserted, causing a duplicate period “..” and resulting in task failure.
Waveform/Parameter Setting	80%	(Task 4) The terminology used in P2 was unfamiliar to the user, leading to task failure.
General Setting	73%	(Task 11) Task failure occurred due to performing the function in service mode.
Alarm	90%	(Task 18) The terminology used in P1 was unfamiliar to the user, leading to task failure.
Display Mode	93%	(Task 42) Task failure occurred due to confusion between the functionalities required in the event review screen and the event review settings screen.
Patient Discharge	100%	-
Total	86%	

**Table 7:** Satisfaction evaluation results for each scenario in the formative evaluation (usability test).

Use Scenario	Mean	SD
Patient Admit	5.00	1.25
Waveform/Parameter Setting	5.73	0.88
General Setting	5.20	1.20
Alarm	5.35	0.82
Display Mode	4.96	1.28
Patient Discharge	7.00	0.00
Total	5.49	0.88

**Table 8:** Sociodemographic characteristics and experience of the summative evaluation participants.

Variable		Usability Test
Age	20–29 years	12
	30–39 years	10
	40–49 years	1
Affiliated hospital	Severance Hospital	23
Work experience	Less than 3 years	9
	More than 3 years, less than 5 years	7
	More than 5 years, less than 10 years	4
	More than 10 years	3
Use experience with similar devices	Less than 3 years	9
	More than 3 years, less than 5 years	7
	More than 5 years, less than 10 years	3
	More than 10 years	4
Manufacturer name (Repetition is acceptable)	Mediana	7
	Phillips	18
	GE	1

**Table 9:** Summative evaluation results for each scenario in the summative evaluation (usability test).

Use Scenario	Success Rate	Satisfaction Evaluation	
		Mean	SD
Patient Admit	98%	4.68	0.63
Waveform/Parameter Setting	87%	4.52	0.65
General Setting	87%	4.48	0.67
Alarm	89%	4.22	0.87
Display Mode	95%	4.47	0.79
Wave Freeze	74%	4.59	0.60
EWS	82%	3.95	1.00
Standby Mode	100%	4.91	0.28
Patient Discharge	100%	5.00	1.00
Total	90%	4.54	0.72

**Table 10:** Statistical analysis of success rates in formative and summative evaluations.

Use Scenario	Mann–Whitney U ^1^	Z	*p*-Value
Patient Admit	37	−2.294	0.239
Waveform/Parameter Setting	27	−1.887	0.071
General Setting	41	−1.140	0.348
Alarm	41.5	−1.009	0.348
Display Mode	50.5	−0.533	0.684
Patient Discharge	57	0.000	1

^1^ Because the data were not normally distributed, the Mann–Whitney U test was performed.

**Table 11:** Statistical analysis of satisfaction scores in formative and summative evaluations.

Use Scenario	Mann–Whitney U ^1^	Z	*p*-Value
Patient Admit	6	−3.314	0.001
Waveform/Parameter Setting	21	−2.204	0.027
General Setting	20	−2.313	0.023
Alarm	31	−1.593	0.121
Display Mode	24	−2.097	0.045
Patient Discharge	57.5	0.000	1.000

^1^ Because the data were not normally distributed, the Mann–Whitney U test was performed.

## Data Availability

Data are available upon demand.

## Supplementary Materials

The following supporting information can be downloaded at: https://www.mdpi.com/article/10.3390/jcm14093218/s1, Table S1: Formative evaluation usability test: survey list; Table S2: Summative evaluation usability test: survey list.

## Abbreviations

The following abbreviations are used in this manuscript:

EWS	Early Warning Score
NEWS	National Early Warning Score
PEWS	Pediatric Early Warning Score
MEWS	Modified Early Warning Score
ICU	Intensive Care Unit
CCU	Cardiac Intensive Care Unit
FDA	Food and Drug Administration
UI	User Interface
ABP	Arterial Blood Pressure
CVP	Central Venous Pressure
