# Supplementary material for: Enhancing the Usability of Patient Monitoring Devices in Intensive Care Units: Usability Engineering Processes for Early Warning System (EWS) Evaluation and Design
Source: J Clin Med. 2025 May 6;14(9):3218. doi: 10.3390/jcm14093218 (PMC12072364; doi:10.3390/jcm14093218)
Supplement: Supplementary file 1 [file jcm-14-03218-s001.zip › jcm-3576786-supplementary.pdf]

Supplementary Table

** 1 : Very Dissatisfied, 7: Very Satisfied*

Table S1. Formative evaluation usability test : survey list

Use Scenario	No	Survey evaluation items	1	2	3	4	5	6	7
**Patient Admit**	1	Do you find the patient registration process to be easy to perform?							
	2	Do you find the process of editing patient information to be easy?							
**Waveform/ Parameter Setting**	3	Do you find it easy to configure the number of displayed waveforms?							
	4	Do you find it easy to modify the label of the IBP waveform?							
	5	Do you find it easy to change the type of waveform being displayed?							
	6	Do you find it easy to adjust the ECG waveform setting?							
	7	Do you find it easy to change the NIBP settings, such as the automatic measurement interval?							
	8	Do you find it easy to modify the respiration settings, including the measurement source?							
**General Settings**	9	Do you find the automatic window closing function and its configuration method easy to use?							
	10	Do you find the audio-related settings easy to configure							
**Alarm**	11	Do you find the location and presentation format of visual alarm indicators to be useful?							
	12	Do you find the function and configuration method for modifying alarm thresholds easy to use?							
	13	Do you find the function and accessibility of the audio alarm mute button for each parameter to be user-friendly?							
	14	Do you find the alarm configuration method in the arrhythmia settings menu easy to use?							
	15	Are you satisfied with the visual and auditory alarm representation according to the severity of arrhythmias?							
	16	Are you satisfied with the operation of the pause button based on arrhythmia severity levels?							
	17	Are you satisfied with the triggering conditions for each type of PVC and the duration of alarm message display?							
	18	Do you find the function and accessibility of the global audio alarm off button easy to use?							

Use Scenario	No	Survey evaluation items	1	2	3	4	5	6	7
**Display Mode**	19	Do you find the default screen layout convenient and effective for operation?							
	20	Are you satisfied with the screen layout that prominently displays numerical values?							
	21	Did you experience any inconvenience with the tabular trend display screen?							
	22	Did you experience any inconvenience with the graphical trend display screen?							
	23	Did you experience any inconvenience with the event review screen?							
**Patient Discharge**	24	Do you find the patient discharge process easy to perform?							

** 1 : Very Dissatisfied, 5: Very Satisfied*

Table S2. Summative evaluation usability test : survey list

Use Scenario	No	Survey evaluation items	1	2	3	4	5
**Patient Admit**	1	Do you find the patient registration process to be easy to perform?					
	2	Do you find the process of editing patient information to be easy?					
**Waveform/ Parameter Setting**	3	Do you find it easy to configure the number of displayed waveforms?					
	4	Do you find it easy to change the waveform display settings, including the ON/OFF functionality?					
	5	Do you find it easy to switch between different waveform types?					
	6	Do you find it easy to adjust ECG waveform settings such as sweep speed and amplitude?					
	7	Do you find it easy to change the NIBP settings, such as the automatic measurement interval?					
	8	Do you find it easy to modify the respiration settings, including the measurement source?					
	9	Do you find the filter mode adjustment within the respiration settings to be useful?					
	10	Do you find it easy to toggle CVP and $EtCO_2$ parameters and waveforms ON or OFF from the display menu?					
**General Settings**	11	Do you find the automatic window closing function and its configuration method easy to use?					
	12	Do you find the audio-related settings easy to configure					
**Alarm**	13	Do you find the location and presentation format of visual alarm indicators to be useful?					
	14	Do you find the function and configuration method for modifying alarm thresholds easy to use?					

Use Scenario	No	Survey evaluation items	1	2	3	4	5
	15	Do you find the function and accessibility of the audio alarm mute button for each parameter to be user-friendly?					
	16	Is the placement of the message list intuitive and user-friendly?					
	17	Do you find the alarm configuration method in the arrhythmia settings menu easy to use?					
	18	Are you satisfied with the visual and auditory alarm representation according to the severity of arrhythmias?					
	19	Are you satisfied with the operation of the pause button based on arrhythmia severity levels?					
	20	Are you satisfied with the triggering conditions for each type of PVC and the duration of alarm message display?					
	21	Do you find the function and accessibility of the global audio alarm off button easy to use?					
**Display Mode**	22	Do you find the default screen layout convenient and effective for operation?					
	23	Are you satisfied with the screen layout that prominently displays numerical values?					
	24	Did you experience any inconvenience with the tabular trend display screen?					
	25	Did you experience any inconvenience with the graphical trend display screen?					
	26	Did you experience any inconvenience with the event review screen?					
**Wave Freeze**	27	Do you find the time interval of the Freeze mode appropriate?					
	28	Was the function of the Wave Freeze button easy to understand?					
**Early Warning Score (EWS)**	29	Do you find the Early Warning Score (EWS) function useful?					
	30	Do you find the EWS trend configuration options (e.g., NEWS, NEWS2, Custom) to be useful?					
	31	Are you satisfied with the message display method shown during total score calculation?					
**Early Warning Score (EWS)**	32	Are you satisfied with how the total score is presented upon calculation?					
	33	Are you satisfied with the visual representation of trend data?					
	34	Are you satisfied with the ON/OFF pop-up setting interface for the EWS menu?					

Use Scenario	No	Survey evaluation items	1	2	3	4	5
**Standby Mode**	35	Do you consider the functionality of the standby mode to be useful in clinical practice?					
**Patient Discharge**	36	Do you find the patient discharge process easy to perform?					
